# Simulation training of caregivers at hospital discharge of patients with chronic diseases: an integrative review

**DOI:** 10.1590/0034-7167-2023-0043

**Published:** 2023-12-04

**Authors:** Luciana Aparecida da Cunha Borges, Rodrigo Guimarães dos Santos Almeida, Elton Santo Barboza, Guilherme Oliveira de Arruda

**Affiliations:** I Universidade Federal de Mato Grosso do Sul. Campo Grande, Mato Grosso do Sul, Brazil; II Universidade Federal do Mato Grosso do Sul. Coxim, Mato Grosso do Sul, Brazil

**Keywords:** Simulation Training, Caregivers, Patient Discharge, Chronic Disease, Nursing, Entrenamiento Simulado, Cuidadores, Alta del Paciente, Enfermedad Crónica, Enfermería, Treinamento por Simulação, Cuidadores, Alta do paciente, Doenças Crônicas, Enfermagem

## Abstract

**Objective:**

to identify evidence about the use and effects of clinical simulation for preparing caregivers for discharging patients with chronic conditions.

**Methods:**

an integrative peer review in the Scopus, PubMed, Web of Science, Cumulative Index to Nursing and Allied Health Literature, ScienceDirect and Virtual Health Library databases, from July to September 2022.

**Results:**

3,218 studies were identified, with a final sample consisting of four national and two international articles. Using simulation as an educational technology contributed to caregiver preparation in home care. In most studies, using clinical simulation included using other strategies to complement training: expository dialogued class, conversation circle and audiovisual resources.

**Final considerations:**

simulation proved to be efficient for training caregivers, with the active participation of family members and nurses in health education actions.

## RESUMO

**Objetivo:**

identificar evidências acerca do uso e dos efeitos da simulação clínica para o preparo de cuidadores na desospitalização de pacientes em condições crônicas.

**Métodos:**

revisão integrativa, realizada por pares, nas bases Scopus, PubMed, *Web of Science, Cumulative Index to Nursing and Allied Health Literature, ScienceDirect* e Biblioteca Virtual de Saúde, no período de julho a setembro de 2022.

**Resultados:**

foram identificados 3.218 estudos, com amostra final composta por quatro artigos nacionais e dois internacionais. O uso da simulação como tecnologia educacional contribuiu para o preparo de cuidadores nos cuidados domiciliares. Na maioria dos estudos, o uso da simulação clínica contou com a utilização de outras estratégias para complemento da formação: aula expositiva dialogada, roda de conversa e recursos audiovisuais.

**Considerações finais:**

a simulação se mostrou eficiente para o treinamento dos cuidadores, com participação ativa de familiares e enfermeiros nas ações de educação em saúde.

## RESUMEN

**Objetivo:**

identificar evidencia sobre el uso y efectos de la simulación clínica para la preparación de cuidadores en la deshospitalización de pacientes con enfermedades crónicas.

**Métodos:**

revisión integradora por pares en Scopus, PubMed, Web of Science, Cumulative Index to Nursing and Allied Health Literature, ScienceDirect y Virtual Health Library, de julio a septiembre de 2022.

**Resultados:**

se identificaron 3.218 estudios, con una muestra final compuesta por cuatro artículos nacionales y dos internacionales. El uso de la simulación como tecnología educativa contribuyó a la preparación de los cuidadores en el cuidado domiciliario. En la mayoría de los estudios, el uso de la simulación clínica incluyó el uso de otras estrategias para complementar la formación: clase expositiva dialogada, círculo de conversación y recursos audiovisuales.

**Consideraciones finales:**

la simulación demostró ser eficiente para la formación de cuidadores, con la participación activa de familiares y enfermeros en acciones de educación en salud.

## INTRODUCTION

Chronic non-communicable diseases affect populations all over the world, but their prevalence is higher in underdeveloped or developing countries, becoming the main cause of morbidity and mortality in all age groups. Challenges faced by patients with chronic illnesses include the need to achieve: relief and management of signs and symptoms; psychological adjustment; physical accommodation and resulting disability; prevention and management of crises and complications; management of family functionality; establishment of support networks and resources that can increase quality of life, among others^(1-2)^.

Faced with these challenges, continuous search for health services to achieve safe care requires those involved in the process, i.e., professionals, patients, family members and their caregivers, to have a comprehensive and collaborative look in the planning and execution of this care. The Global Patient Safety Action Plan (2021-2030), in its guiding principles, emphasizes the importance of involving the health team in patient care, especially in the transition from care in hospital institutions to home care during discharge^(3)^.

Discharge occurs when patients, still hospitalized, have a satisfactory clinical stability of their health status, but will need to complement treatment at home, which may include professional follow-up, use of life-supporting equipment and attention of a caregiver^(4)^. In this regard, it is considered important that informal caregivers are prepared even in a hospital environment so that they feel safer, more confident and capable of providing home care. Evidence produced in a quasi-experimental study denote that caregiver preparation in the hospital, through guidance and training, combined with post-discharge follow-up, increases the skills of this caregiver and reduces the chances of inappropriate actions at home^(5)^. In order to comply with the Patient Safety Policy in the discharge process, a teaching and learning strategy called “clinical simulation in health” has been highlighted. It is defined as an educational strategy that exposes participants to hypothetical scenarios that mimic the reality of clinical practice^(6)^. Clinical simulation practice is common for students, during academic training, and also for health professionals, in learning and improving health care, being a technology that has been improved in recent times for teaching and learning in several areas^(7)^. However, it is still an uncommon practice in interventions to prepare caregivers for discharge.

For this strategy to work, its phases, called preparation, participation and debriefing, must be conceptualized and understood. The preparation phase is divided into two stages: a pre-simulation, which covers participant preparation for the topics presented in clinical simulation, through the delivery of educational materials and skills training; and a pre-briefing/briefing, set up by interaction between facilitator and participant, immediately in the field, with clear scenarios, objectives and learning roles^(8)^.

The participation phase involves carrying out the proposed scenario, while debriefing presents a discussion/reflection process that takes place during or after the scenario, enabling the improvement of skills and abilities. Clinical simulation planned and executed in three stages enables the teaching and learning process of complex subjects^(8)^.

Patientand family-centered clinical simulation presents many challenges for academic and professional education. The financial, structural and organizational challenges in the clinical simulation proposal aimed at home care education include, in addition to caregivers’ literacy level, their feelings, emotional and affective states, which influence learning^(9)^.

This study was developed because it is understood that clinical simulation can also be developed with caregivers, with a view to producing safe home care, and because it was verified that the aforementioned teaching and learning methodology appears little among the interventions developed with caregivers in the discharge process.

## OBJECTIVE

To identify evidence about the use and effects of clinical simulation to prepare caregivers for discharging patients with chronic conditions.

## METHODS

### Ethical aspects

According to Resolution 466/2012 of the Brazilian National Health Council of the Ministry of Health, as it is a review study, appraisal by an Ethics Committee is waived.

### Study design

This is an integrative literature review, which included the following steps: guiding question elaboration; establishment of inclusion and exclusion criteria; search or sampling in the literature; data collect; categorization of studies; critical analysis of included studies; discussion of results; and integrative review presentation^(10-11)^.

The theme “clinical simulation in preparation for discharge” was established. From there, the object of the study was defined, specifying the interest in preparing caregivers of people with chronic conditions and, consequently, elaborating the guiding question: how is clinical simulation used and what are its effects on preparation of caregivers of patients with chronic conditions in the discharge process? The acronym PVO was used, from which the following attributions were performed: P (population): caregivers of patients with chronic diseases; V (variable): clinical simulation; and O (outcome): discharge.

### Study period and place

The search for articles in the databases took place in July 2022. Two independent researchers searched for the studies, in pairs. It is noteworthy that search strategies were defined after mapping the terms and synonyms searched in the Medical Subject Headings (MeSH) and Descriptors of Health Sciences (DeCS). The terms, in Portuguese and English, were combined in different ways, using the Boolean operators “OR” and “AND”. For testing with the broader strategy, a search was also carried out for the most used words in titles and descriptors/keywords of research already published on the subject. To search the bases, the indexed descriptors and their respective synonyms in MeSH were used (Chart 1).

**Chart 1:** Indexed descriptors and their respective synonyms in Medical Subject Headings, 2022

Acronym	Meaning	Terms
**P**	Population	**1#** (“Caregivers” OR “Caregiver” OR “Carers” OR “Carer” OR “Care Givers” OR “Care Giver” OR “Spouse Caregivers” OR “Caregiver, Spouse” OR “Caregivers, Spouse” OR “Spouse Caregiver” OR “Family Caregivers” OR “Caregiver, Family” OR “Caregivers, Family” OR “Family Caregiver” OR “Informal Caregivers” OR “Caregiver, Informal” OR “Caregivers, Informal” OR “Informal Caregiver”) **2#** (“Chronic Disease” OR “Disease, Chronic” OR “Diseases, Chronic” OR “Chronic Illness” OR “Chronic Illnesses” OR “Illness, Chronic” OR “Illnesses, Chronic” OR “Chronically Ill”)
**V**	Variable	**3#** (“Simulation Training” OR “Training, Simulation” OR “Interactive Learning” OR “Learning, Interactive” OR “Simulation-based education” OR “Simulation based education” OR “Simulation” OR “Patient simulations” OR “Patient Simulations” OR “Simulation, Patient” OR “Simulations, Patient”)
**O**	Outcome	**4#** (“Transitional Care” OR “Care, Transitional” OR “Cares, Transitional” OR “Transitional Cares” OR “Transition Care” OR “Transition Cares” OR “Home Transition” OR “Home Transitions” OR “Transition, Home” OR “Transitions, Home” OR “Patient Discharge” OR “Discharge, Patient” OR “Discharges, Patient” OR “Patient Discharges” OR “Discharge Planning” OR “Discharge Plannings” OR “Planning, Discharge” OR “Plannings, Discharge” OR “De-hospitalization”) and an uncontrolled descriptor in MeSH

It is noteworthy that synonyms were used in order to identify the largest possible number of publications related to the object under study. Database crossings occurred with the use of the AND operator, namely: crossing 1: “Caregivers AND Chronic disease AND Simulation Training AND Transitional Care”; crossing 2: “Caregivers AND Simulation Training AND Transitional Care”; crossing 3: “Simulation Training AND Transitional Care”. The awareness strategies used are shown in Chart 1. Due to specificities and for the feasibility of the review, different strategies were used for each base.

This review was carried out in the following indexed databases: Scopus; National Library of Medicine (PubMed); Web of Science; ScienceDirect; Cumulative Index to Nursing and Allied Health Literature (CINAHL); and Virtual Health Library (VHL) portal. The analysis of collected material was carried out in September 2022.

### Population and sample

A total of 3,218 studies were found in the selected data sources. After removal of paid materials and exclusion based on initial reading of titles, abstracts and removal of duplicates, six studies were selected to compose the results.

### Inclusion and exclusion criteria

Inclusion and exclusion criteria were defined, including complete articles available in full, online and free of charge that answered the guiding question, in addition to studies in any language and without time frame. Editorials, letters to the editor, abstracts, expert opinion, other reviews, correspondence, reviews, book chapters, monographs, theses and dissertations as well as duplicate productions were excluded.

### Study protocol

All databases were accessed via the Federated Academic Community (CAFe - *Comunidade Acadêmica Federada*), in the Coordination for the Improvement of Higher Education Personnel (CAPES - *Coordenação de Aperfeiçoamento de Pessoal de Nível Superior*) Journal Portal. After searching for studies in the databases, the articles were exported to the Zotero 5.0 reference manager, where duplicates were excluded. Study selection and analysis were carried out by two researchers, master’s students of the graduate course in nursing at the Integrated Health Institute of the *Universidade Federal do Mato Grosso do Sul*, members of research groups linked to the aforementioned program. When there were disagreements, a third reviewer, professor and leader of the research group, was contacted, minimizing biases regarding selection and interpretation errors.

### Analysis of results

After defining and extracting information from the articles, a chart was prepared in Microsoft Excel 2013, containing study identification, reference, objective, methodological approach, type of simulation and simulators used, impact factor of the journal in which the article was published, levels of evidence and main results.

For the critical assessment of the studies included in the review, which included hierarchical classification regarding the level of evidence, the primary study’s research question was considered. For this assessment, three types of questions and the levels of evidence corresponding to them were proposed, namely: 1: treatment or intervention (seven levels of evidence); 2: prognosis or etiology (five levels of evidence); and 3: meaning or experience (five levels of evidence)^(12)^.

In the corpus of this review, the classification of evidence from studies with the clinical question directed to treatment or intervention in the health area was used, according to the following hierarchy: N1: systematic review or meta-analysis of randomized controlled clinical trials; N2: randomized controlled clinical trials; N3: clinical trials without randomization; N4: cohort and case-control; N5: systematic review of descriptive and qualitative studies; N6: descriptive or qualitative study; N7: expert opinion^(12)^.

It should be added that the information analyzed in the primary research included in this study was presented unchanged, as per the original production. They were only translated into Portuguese when the article was in Spanish or English.

## RESULTS

**Figure 1:**
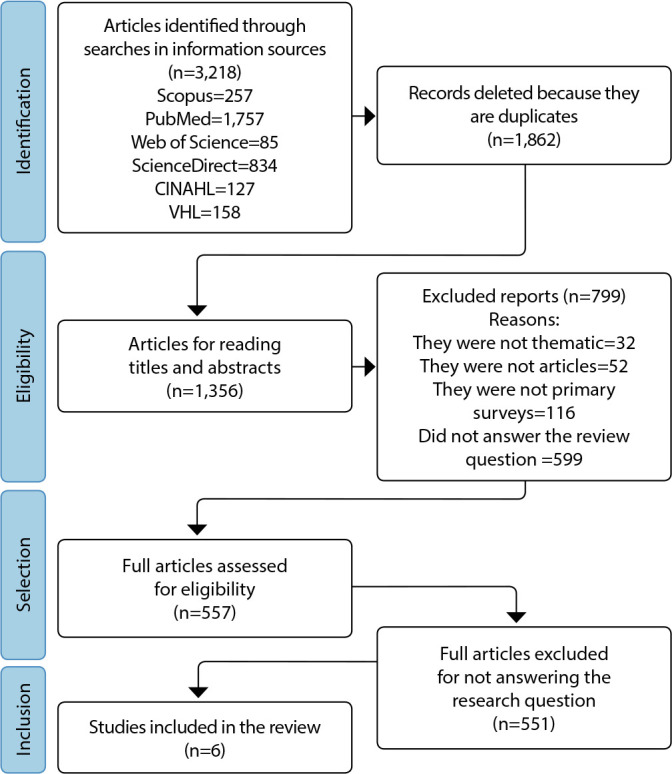
Flowchart of identification, selection and inclusion of studies, prepared from the Preferred Reporting Items for Systematic Reviews and Meta-Analyses (PRISMA) recommendation, 2022

The sample of this review study comprises six articles. Figure 1 shows the article selection route flowchart.

Thereafter, the characterization of selected studies will be described. When analyzing the period of publications, according to triennial distribution, from 2012 to 2014, an article was published; from 2018 to 2020, four articles; and in 2022, an article. Regarding study origin, four were produced in Brazil and two in the United States. With regard to the methodological approach, two studies with a qualitative approach and four studies with a quantitative approach were found. With regard to the studies’ level of evidence, two were level VI and four were level IV.

Chart 2 shows the numbering according to reference, authors, year of publication, author training, article title, journal name, study origin, impact factor and level of evidence.

**Chart 2:** Characterization of articles regarding authors, year of publication, author training, article title, journal name, country of publication, impact factor and level of evidence, 2022

ID	Authors and year of publication	Author training	Article title	Journal name and country of publication	IF	LoE
A1	Silva M, Charlo PB, Zulin A, Santos FGT, Jaques AE, Haddad MCFL, Radovanovic CAT. (2022)^(13)^	Nurses and physician	Construction and validation of clinical scenarios for training informal caregivers of dependent people	*Revista Brasileira de Enfermagem* - REBEn Brazil	0.705	IV
A2	Silva APM, Pina JC, Rocha PK, Anders JC, Souza AIJ, Okido ACC. (2020)^(14)^	Nurses	Training of caregivers of children with special health needs: simulation contributions	*Texto & Contexto - Enfermagem* Brazil	0.589	VI
A3	Santos AST, Góes FGB, Ledo BC, Silva LF, Bastos MPC, Silva MA. (2020) ^(15)^	Nurses	Educational technology on home care for low-risk newborns	UERJ Nursing Journal Brazil	None	VI
A4	Stanley TA, Battles M, Bezruczko N, Latty C. (2019)^(16)^	Nurses and psychologist	Simulation effectiveness for caregivers of children with tracheostomy	Clinical Simulation in Nursing United States	2.391	IV
A5	Thrasher J, Baker J, Ventre KM, Martin SE, Dawson J, Cox R, Moore HM, Brethouwer S, Sables-Baus S, Baker CD. (2018^)(17)^	Physicians	From hospital to home: A quality improvement initiative to implement high-fidelity simulation training for caregivers of children requiring long-term mechanical ventilation	Journal of Pediatric Nursing United States	2.145	IV
A6	Esteves MT, De Domencio EBL, Petito EL, Gutiérrez MGR. (2013)^(18)^	Nurses and physical therapist	Educational intervention for self-monitoring of continuous drainage in the postoperative period of mastectomy	*Revista Gaúcha de Enfermagem* Brazil	0.638	IV

*Caption: ID - - identification; IF - impact factor; LoE - level of evidence, classified according to clinical questions of treatment or intervention.*

As for the format, simulations were predominantly developed in a skills training format. A1 used low and medium fidelity training; A2, A3 and A5 used the low-fidelity one; eA4 used high-fidelity training to prepare family caregivers. Chart 3 presents the objectives, method, participants, types of simulation and simulators, main results and limitations of the studies.

**Chart 3:** Characterization of articles regarding objective, method, participants, type of simulation and simulator of the articles included in this review, 2022

ID	Objective	Method	Participants	Type of simulation and simulator
A1	Build and validate three clinical scenarios for training informal caregivers of dependent people.	Methodological study, developed in two stages: construction of scenarios; and content validity by experts.	12 experts in active learning technologies and/or care for dependent patients.	Scenario construction (patient hygiene and comfort; bed bath, skin hydration and change of position); simulated patients (actors)
A2	Know the contributions of simulation to training caregivers of children with special health needs in preparation for hospital discharge.	Descriptive-exploratory study with a qualitative approach	15 caregivers of children with special health needs (CHISHN).	Skills training; low, medium and high-fidelity pediatric simulators
A3	Identify doubts of postpartum women and family members about home care for low-risk newborns; analyze the conversation circle, mediated by a realistic low-fidelity simulator, as an educational technology to prepare families in the maternity discharge process.	Qualitative study	19 family members of low-risk newborns in a municipal hospital in Rio das Ostras, Rio de Janeiro.	Realistic low-fidelity simulator; static vinyl mannequin with dimensions similar to a newborn’s body, fitted with a fictitious plastic umbilical stump and a clamp attached to it
A4	Show that simulation improves confidence and competence in caregivers of children with a tracheostomy and to examine the impacts on emergency room visits and mortality.	All participants received standard classroom training. One group also received simulation training.	Caregivers (parents) of children who received a new tracheostomy.	High-fidelity simulation; human simulator
A5	Create a multimodal discharge readiness curriculum incorporating high-fidelity simulation training to prepare family caregivers of children with complex physicians, conditions that require long-term mechanical ventilation.	Methodological study with a qualitative approach	Family caregivers	High-fidelity simulation; simulator; PediaSIM ECS (CAE Healthcare; Montreal, QC Canada). The simulator is a computer-controlled mannequin.
A6	Assess the performance of patients undergoing surgery for breast cancer in self-monitoring of continuous drainage system.	Prospective study of educational intervention	79 women who underwent surgery for breast cancer and had a drain (continuous drainage system).	Classroom and simulation of continuous drainage system management, performance assessment and reinforcement of guidelines; low-fidelity simulator

*Caption: ID - identification.*

As for study objectives, one was developed for clinical reasoning training, and five for skills training. The findings indicate positive effects regarding the use of simulation as a teaching strategy, in which it improves the performance of caregivers trained in a simulated environment, improvement of technical skills, in addition to development of skills required for patient care stand out.

With regard to the population, three studies addressed simulation in preparation for caring for children^(14,16-17)^, one with newborns^(15)^, one with mastectomized women^(18)^, and one with informal caregivers of dependent people^(13)^.

Simulation through skills training was mainly used for training and health education aimed at family caregivers of children with special needs using tracheostomy, using long-term mechanical ventilation, in the process of discharge from maternity hospital to home and, finally, in the self-monitoring of continuous drainage system. It was developed using audiovisual materials, a training course at the simulation center and a conversation circle, in addition to a dialogued expository class. The characterization of the articles included in this integrative review is represented in Charts 3 and 4.

**Chart 4:** Characterization of articles regarding main results and limitations of the articles included in this review, 2022

ID	Main results	Limitations
A1	Simulation scenarios proved to be appropriate, obtaining an average value of 91.6%. However, some adjustments were made to its organization regarding clarity in the wording of guidelines, as suggested by judges.	The study used only three scenarios; therefore, it was limited by the impossibility of including other important themes in the script. Inserting new themes, alluding to bed bath practices for dependent people at home, would make the video significantly longer in duration, which could harm the main objective and cause the target audience to have less assimilation of the theme addressed. Another limiting issue refers to the need for assessment by the target audience to make the material more cohesive and applicable, in order to configure the realism necessary for the video to have a positive impact on educational issues.
A2	Two categories emerged: Simulation experience as a learning strategy; and Implications of training for home care. Simulations allowed the improvement of procedural skills and coping with possible intercurrences at home. Initially, feelings of fear and anxiety were triggered, especially in high-fidelity simulation. However, after training, caregivers felt relieved, self-confident and satisfied with their performance, highlighting the importance of the support provided during simulations. They mentioned greater confidence in carrying out the procedures on the child and in facing the challenges of home care.	They are limited to technology-dependent children, who represent only a subgroup of CHISHN. Only caregivers of children using oxygen therapy, tracheostomy, gastrostomy and nasojejunal tube participated.
A3	The family members’ doubts were about care with hygiene, food, environment, affection, health, sleep and illnesses. Conversation circle with a low-fidelity simulator was considered a positive strategy to mediate learning.	The limitation of this study refers to the single geographical context, requiring further studies in different social realities, in addition to investigations that validate this educational technology in a representative sample of the target audience.
A4	Post-test confidence was significantly higher in the simulation group. The correlation between post-test confidence and competence measures was not statistically significant. Mortality in family homes was nearly five times higher for children whose caregivers did not participate in simulation.	None.
A5	A total of 87 family caregivers completed the curriculum. This simulation-enhanced curriculum was well received by participants. They reported that post-simulation debriefing was the most beneficial component. There was a trend towards a reduction in readmissions within seven days of implementing the revised curriculum.	First, we limited the scope of the project by offering simulation-based curriculum only to families of children requiring long-term mechanical ventilation. The institution’s inventory required the researchers to use a manikin representing a 5-year-old child, whereas patients’ mean age was 11.5 months. The research was conducted in a single center, and it is not known whether the findings are generalizable to other settings.
A6	It is believed that the combination of educational strategies capable of generating the acquisition of new skills, such as simulation and control of self-management of the drainage system at home, provided a favorable result for drain obstruction prevention, noting that 84.8% kept the system patent. It was found that training focused on self-care exercised by patients and the strategy used favorably influenced self-monitoring of continuous drainage system, preventing drain obstruction, a fact evidenced by the percentage of patients who maintained system patency (84.2%).	None.

*Caption: ID - identification.*

## DISCUSSION

This review sought to identify evidence about the use and effects of simulated clinical practice with caregivers of patients with chronic health conditions as a training strategy in preparing patients for discharge. The evidence found reflects that use of simulation as a teaching and training strategy has been of international and national relevance^(19)^, allowing caregivers to have contact with real contexts simulated in home discharge care, which provides security for caregivers so that they are able to develop not only technical skills, but also clinical reasoning in emergency situations that may cause damage to the real patient in both hospital and home settings. Thus, simulation proved to be a significant learning tool^(14-15)^ in preparing caregivers to act in critical conditions.

In A1, simulated patients were used to train caregivers through educational videos. Moreover, most articles that addressed simulation as a training technique (A2, A3, A4 and A5) in caregiver preparation were published in the last 5 years, and only one article (A6) contemplated in the review was published longer than this period. This demonstrates the relevance of the discussion today.

The articles included in this integrative review highlighted the importance of carrying out educational actions during hospitalization as a strategy to train family and caregiver for the care to be performed at home after hospital discharge.

Therefore, simulation can be seen as a strategy that provides the opportunity to provide safe and controlled care in a practice similar to what will be done at home, thus promoting safe patient care^(20-21)^. Thus, it is necessary for caregivers to be guided and trained, in a more reliable way, to provide adequate home care for users dependent on care^(13)^.

The use of simulated scenarios as an educational tool has shown promise, and there is positive evidence that it can be used to prevent adverse events arising from inadequate care^(22-23)^.

Moreover, in the case of caregivers who receive training to face the limitations of the health condition of users undergoing treatment, their skills and care practices become adequate, resolute and precise, which culminates in the reduction of stressful events related to care^(24-25)^.

That said, A1 brings the relevance of implementing a methodology using information technology, building realistic clinical simulation scenarios through educational videos; this would be an important tool in caregivers’ educational process and training. These strategies can directly impact users’ quality of life and reduce the need for health care^(26-28)^.

In A4, caregivers, parents of children with tracheostomy, expressed confidence and security after training with the use of a mannequin and simulated patient, and training was directed to the needs of each child and family. A decrease in the mortality rate was also observed in the homes of family members of caregivers who participated in simulations. Participants reported acquiring empowerment and greater preparation for care after hospital discharge, preventing home complications from complicating patients’ clinical condition^(16)^.

Confidence and care competence increased with participation in simulation training, with caregivers becoming, over time, more sensitive to their children’s suffering and more likely to use the emergency service when deemed necessary by parents who participated in the research. The predominant expectation in the research was that skills and confidence converged as caregivers gained experience^(16)^.

The results, therefore, revealed that simulation practice has been shown to be more effective for teaching and learning than traditional teaching. Simulation is a strategy that allows a greater practical experience according to reality, collaborating for a more risk-free assistance^(29)^. A2, A3, A4, A5 and A6 caregivers considered that training contributed to safety in carrying out care and procedures provided to patients, thus providing support in continuity of treatment at home and in facing the challenges of home care, avoiding readmissions.

Through in-depth reading of the articles that make up the *corpus* of this review, it is clear that simulation is an educational and innovative tool in training caregivers in the face of complex care. It is a practice capable of improving caregivers’ and family members’ skills and knowledge in the exchange of clinical experience, contributing to qualifying home care as well as encouraging empowerment and self-confidence^(14-15)^.

The studies found had the participation of different professionals who make up the multidisciplinary health team, such as nurses, physicians, physiotherapists and psychologists, demonstrating that professionals are committed to improving their performance and the relationship with the team^(30)^. It is noteworthy that most of professionals in the studies were nurses.

A2, whose objective was to know the simulation strategies for training caregivers of children with special health needs in preparation for hospital discharge, showed that professional nurses played the social role of educator, equipping families with home care. This professional represented an important element in preparing these patients for hospital discharge. Knowledge was transmitted through demonstration, in which skills were improved for the execution of techniques. As caregivers need to be prepared to deal with possible complications at home, there is a need for strategies that favor the role of caregivers in the process of caring for patients^(14,31)^.

Regarding the types of simulations and simulators found in the articles, a strategy addressed by the researchers was conversation circle with low-fidelity simulation as an educational technology, focusing on participants’ doubts and the use of a static vinyl mannequin with dimensions of a newborn body. This strategy proved to be effective in meeting the family’s learning needs in the transition from motherhood to home. After its completion, the educational action received positive feedback from caregivers: everyone considered it efficient and valid in understanding baby care^(14)^.

It stands out as a valid strategy for training caregivers, as it seeks to explore the potential of simulation in a context different from the one in which it will be used, which is the training of family caregivers involved in home care. This is an innovative study, as it sought to qualify home care through the use of simulation. In this context, two categories of feelings emerged: Experience of simulation as a learning strategy; and Implications of training for home care^(14)^.

Research carried out in a simulation center of a public university in southern Brazil developed a training program based on simulations to prepare caregivers of technology-dependent children for hospital discharge. As a health education strategy associated with low-fidelity simulation, a conversation circle was held in order to demonstrate procedural techniques, such as the use of a dummy with technological devices. The findings showed positive results, as long as simulation is performed procedurally during hospitalization, as it favored the exchange of knowledge and experiences among participants, in addition to promoting safety in care^(32)^.

A2 used simulation strategies associated with low, medium and high-fidelity simulators, in a systematic way, in preparing caregivers to face possible complications at home^(14)^. Adequate preparation of caregivers for hospital discharge is especially important, as it reduces the risk of readmissions due to domestic complications that impact the worsening of children’s health status^(33)^.

According to the North American literature, the period of adaptation of parents to the therapeutic regime can be influenced by the type of procedure to be performed. Procedures that require greater frequency, such as tracheostomy care, were learned more easily, and helped to reduce the chances of readmissions to hospital services. As family members experienced daily child care and developed the necessary skills to care for this device, readmissions became less frequent^(34)^.

Similarly, a North American study (A5), whose objective was to create a multimodal discharge preparation curriculum, incorporating high-fidelity simulation training, to prepare family caregivers of children with complex medical problems in conditions that require mechanical ventilation over the long term, resulted in a trend of reduced readmissions within seven days of discharge since the implementation of the revised curriculum. Simulation training can be incorporated into discharge training for families of children requiring prolonged mechanical ventilation. Trial of emergency management in a simulated clinical setting increased caregiver confidence to take care of their ventilator-dependent child^(17)^.

In a survey whose objective was to analyze the association between outpatient follow-up of children with medical complexities and readmissions in the first 30 days, it was identified that, after hospital discharge, as family members gained experience in daily care necessary for children, their skills were developed through the proper care of this device. Readmissions were also less frequent^(34)^.

Caregiver training, therefore, can have an impact on reducing the deterioration of patients’ clinical condition, avoiding new hospitalizations that, sometimes, can accompany the emergence of new needs^(14)^.

A study that investigated professionals’ knowledge through simulated scenarios revealed that the level of knowledge was low before simulations and that, after implementing training with this strategy, there was a perception of improvement in knowledge, suggesting that simulation should be widely implemented^(35)^.

In this review, the use of simulation in the development of different skills prevailed, both technical (tracheostomy; oxygen therapy; gastrostomy; nasojejunal tube; cardiopulmonary resuscitation; mechanical ventilation; and drain) and non-technical (communication; control of anxiety, fear, insecurity and stress; decision-making; and management and confidence building), related to caregivers’ attitudes, values, and experiences. The literature argues that skills are fundamental and necessary to perform a given task^(36-37)^, i.e., caregivers with greater technical skills represent a differential for success in home care.

The results of a quasi-experimental study that investigated the use of *in situ* simulation training to improve professionals’ skills in a scenario of postpartum hemorrhage showed that simulated training emphasizes mastery and experience, verbal persuasion and physiological state, improving collective skills^(38)^.

A3 showed that clinical simulation, during skills training, is useful for the discharge process and for proposing an innovative educational technology in the context of home care for the main actors in this process: caregivers, the family, and patients who need care for their chronic conditions^(15)^.

Furthermore, simulation-based education plays, every day, an increasingly important role in health education around the world, as it enables the creation of conditions that optimize learning, in addition to protecting users from possible risks^(39)^.

### Study limitations

A possible limitation is the unavailability of some studies in freely accessible databases. Also noteworthy is the number of studies found (only six), which demonstrates that the use of clinical simulation in caregiver training still represents a challenge. Most studies are focused on caregivers of child patients (children and newborns), which indicates a gap for studies involving caregivers of adult patients and patients with chronic health conditions.

### Contributions to nursing, health, or public policies

This study provides possible strategies for training caregivers of chronic patients to be used in clinical nursing practice, helping to develop safer, evidence-based care. It can also help in a better caregiver qualification, through clinical simulation as an effective teaching-learning methodology, considering it a tool for qualifying caregivers in home care.

## FINAL CONSIDERATIONS

The findings of this review indicate that caregivers trained through clinical simulation improved procedural skills and coping with possible complications at home, which led to positive changes in patients’ clinical outcomes, with a reduction in home complications and readmissions, due to the adequate management of patients.

This study made it possible to verify that the simulation strategies performed with caregivers for the discharge process were conducted, for the most part, by professional nurses.

In the synthesis of results, several strategies were found combined with the use of clinical simulation to guide and train caregivers. Among the identified strategies, researchers used conversation circle with a low-fidelity simulator to remove doubts from family members, which dealt with basic life needs, along with procedural skills training, high-fidelity simulation with simulated patients and computer-controlled mannequins, to prepare family caregivers of children with complex medical problems. These educational technologies applied to health constituted important resources to improve health care for patients discharged from hospital or under home care.

It is inferred the importance of considering the rigor for the use of clinical simulation as well as to assess its applicability in practice. Finally, a gap is identified with regard to clinical simulation studies aimed at training caregivers for adult patients.

The findings of this study indicate the importance of simulation as an educational technology for caregivers and family members of patients, especially for the acquisition of skills (knowledge, skills and attitudes) aimed at different health care themes and scenarios. The use of this technology facilitated demonstration of care and the understanding of how to carry it out, favoring the reflection of the situation to be experienced at home from visualization of a concrete environment of care.
